# Effects of vestibular rehabilitation in the management of patients with and without vestibular migraine

**DOI:** 10.1016/j.bjorl.2021.07.011

**Published:** 2021-10-25

**Authors:** Ahmet Koc, Elvan Cevizci Akkılıc

**Affiliations:** a Acıbadem Mehmet Ali Aydınlar University, School of Medicine, Department of ENT, Istanbul, Turkey; b Acıbadem Kozyatagı Hospital, Neurology Department, Istanbul, Turkey

**Keywords:** Vestibular migraine, Vestibular rehabilitation, Vertigo

## Abstract

**Objective:**

Vestibular Migraine (VM) is the second most common cause in patients with vertigo. Patients with VM complain about vestibular symptoms during a headache attack or during the period between attacks. Vestibular Rehabilitation (VR), an exercised based therapy to treat dizziness and balance dysfunction has been shown to be effective in vestibular diseases. In this study, we aimed to assess the effect of VR for vestibular symptoms and quality of life in VM patients, and to compare the results with patients with vestibular disorders without migraine.

**Methods:**

Sixty (60) patients who received VR treatment were divided into two groups: vestibular migraine group (30 patients) and non-migraine vestibular dysfunction group (30 patients). All patients received VR for 18 sessions and the program was completed in 1.5 months. Pre- and post-treatment Dizziness Handicap Inventory (DHI) scores, Vestibular Disorders Activities of Daily Living Scale (VADL) scores, the frequency of dizziness and headache, and Computerized Dynamic Posturography (CDP) scores were assessed and compared retrospectively.

**Results:**

With VR in both the vestibular migraine group and vestibular dysfunction group, DHI score, VADL score, the frequency of dizziness and headache scores significantly impaired. Post-treatment CDP results were higher than pre- treatment results for both patient groups.

**Conclusion:**

With VR, a significant improvement was observed in subjective and objective balance assessment measurement. Vestibular Rehabilitation must be considered in patients who do not benefit from medical therapy or have limited benefit.

**Level of evidence:**

Level III (evidence obtained from well-designed controlled trials without randomization).

## Highlights

• Vestibular Migraine (VM) is the second most common cause after Benign Paroxysmal Positional Vertigo in patients with vertigo.
• Drugs used in migraine headaches are also used in the treatment of VM, but complaints of dizziness/vertigo may progress.
• Vestibular Rehabilitation (VR) provide significant improvement in vestibular symptoms and quality of life in VM patients.
• VR should be considered in addition to medical treatment in patients with vestibular migraine.

## Introduction

Migraine is a primary neurological disease characterized by recurrent headache.^1^ In patients with migraine, vestibular symptoms such as dizziness, vertigo, and unsteadiness are more common than the general population.^2^, ^3^, ^4^ Considering that vestibular symptoms are due to the migraine mechanism; the diagnosis is called Vestibular Migraine (VM). VM is the second most common cause after Benign Paroxysmal Positional Vertigo (BPPV) in many studies evaluating patients with vertigo.^5^, ^6^ Epidemiological studies show the prevalence rate of VM as 2.7%.^7^ Most of the patients are in the middle age group, and it is more common in women. A comprehensive definition of the disease was made by the Barany Society and the International Headache Society in 2012.^5^

In recent years, exercise-based therapy, which is named Vestibular Rehabilitation (VR) or balance retraining, has been used to treat dizziness due to vestibular dysfunction.^8^ VR is a treatment method used to treat dizziness and balance dysfunction, based on central mechanisms of neuroplasticity, and accelerates vestibular compensation.^9^ It has been reported that the vestibular symptoms of dizzy patients with and without migraine reduced with VR.^10^, ^11^, ^12^, ^13^, ^14^, ^15^, ^16^, ^17^

This study aims to assess the efficacy of VR therapy for patients with VM in vestibular symptoms and quality of life and compare the results with patients with vestibular disorders without migraine.

## Methods

Patients who received VR treatment between October 2018 and May 2020 were evaluated retrospectively. The patients included in the study were admitted to the ENT and Neurology clinic with complaints of dizziness/vertigo. Those over 18 years old and those suffering from dizziness/vertigo for at least three months despite medical treatment were selected among the patients. The patients were divided into two groups as the Vestibular Migraine (VM) group and the patients with non-migraine Vestibular Dysfunction (VD) group. The diagnosis of VM was made according to the criteria defined by the Barany Society (BS) and the International Headache Society (IHS).^5^ Diagnosis of the patients in the VD group were vestibular neuritis, Persistent Postural Perception Dizziness (PPPD), and prolonged Benign Paroxysmal Positional Vertigo (BPPV) did not respond to medical therapy. Meniere patients were not included in the study. Otological and neurological examination, Videonystagmography (VNG), and Video Head Impulse Test (VHIT) were performed in all patients. There was no migraine or any other type of headache in the patients included in the non-migraineurs vestibular dysfunction group.

There were 30 patients in the VM Group and 30 patients in the VD Group. The mean age of the patients in the VM Group was 46 ± 13 years (range, 30–64 years), and 2 were men, and 28 were women. The patients’ mean age in the VD Group was 56 ± 50 years (range, 38–76 years), 10 men and 20 women. The average duration of dizziness/vertigo complaints in the VM Group was 9.3 months. In the VD Group, the mean duration was 10.2 months.

All patients received VR provided by two physiotherapists who specialized in VR and were blinded to the subject's group allocation. Computerized Dynamic Posturography (CDP) was performed on patients in both groups after obtaining written consent forms from the patients. The vestibular rehabilitation program was initiated after the CDP. The VR program was implemented as three sessions per week for 18 sessions, and the session duration was approximately one and a half hours. The program was completed in 1.5 months.

To evaluate the effect of VR, the results of pre-treatment and post-treatment Dizziness Handicap Inventory (DHI) scores (a standard questionnaire that quantitatively evaluates the degree of handicap in patients' daily lives with vestibular disorders),^18^ and Vestibular Disorders Activities of Daily Scale (VADL) scores (to evaluate the effects of VR on independence in everyday activities of daily living),^19^ and the frequency of dizziness and headache, and CDP scores were assessed and compared. After the last exercise, dynamic computed posturography was repeated in all patients, and values were measured. This study was approved by the University Ethical Committee (2020–18/05). Informed written consent was obtained from all participants.

In order to evaluate the frequency of dizziness, the number of dizziness in one month were recorded. Frequency of dizziness was rated on a 7-point scale as follows: never or less than once a week (1), once a week (2), two to three times a week (3), four to six times a week (4), once a day (5), more than once a day (6), and always (7).

In order to evaluate the frequency of headache, the number of headaches in one month were recorded. Frequency of headache was rated by 1 item on a 7-point scale as follows: never or less than once a week (1), once a week (2), two to three times a week (3), four to six times a week (4), once a day (5), more than once a day (6), and always (7).

Balance function measured by CDP (Synapsys®, Marseille, France). CDP is an evaluation technique that analyzes sensory, motor, and central injuries in vestibular organs. The Sensory Organization Test (SOT) is a part of CDP, and it defines the visual, vestibular, and somatosensory inputs that the person uses in maintaining balance and identifies the defect that causes imbalance. Six standardized sensory conditions of SOT were applied to all patients in the CDP test: Condition 1: static platform – open vision; Condition 2: static platform – closed vision; Condition 3: static platform – deceptive vision; Condition 4: Unstable platform – open vision; Condition 5: Unstable platform – closed vision; Condition 6: Unstable platform – deceptive vision. According to the SOT results, visual, vestibular, somatosensory, preferential, and global scores were calculated.

CDP results of the patients before and after treatment were compared.

The VR program applied to the patients consisted of two parts. In the first part, exercises were done with Computed Dynamic Posturography (CDP). In the second part, static and dynamic rehabilitation exercises were applied. Rehabilitation exercises with CDP are proprioceptive stimulations (somesthetic and vestibular), visual stimulations, and postural biofeedback exercises (stabilization exercises, weight transfer (shift) exercises, weight-bearing exercises, postural control exercises). VR treatment protocol included strengthening and stretching exercises, gaze stability exercises, habituation exercises, exercises to promote vestibular compensation, balance and gait training, exercises to enhance the use of specific sensory inputs for balance control. Exercise groups are: a) Head movement exercises; b) Gaze stabilization or VOR adaptation exercises; c) Standing and walking balance exercises; d) Posture control exercises: on thromboline; e) Eye stabilization exercises: on the diode bar; f) Hand-eye coordination exercises with the ball; g) Coordination exercises with a pilates ball.

### Statistical analysis

Mean, standard deviation, median, minimum, and maximum value frequency and percentage were used for descriptive statistics. The distribution of variables was checked with Kolmogorov-Simirnov test. Mann-Whitney *U* test was used for the comparison of quantitative data. Wilcoxon test was used for the repeated measurement analysis; *p*-values smaller than 0.05 (<0.05) were considered significant. Statistical Package for the Social Sciences (SPSS) version 27.0 was used for statistical analyses.

## Results

Characteristics of the patients, including age, gender, average vestibular symptom duration, and vestibular test results, are shown in Table 1. There was no significant difference between the two groups in terms of vertiginous symptom durations and vestibular test results.

**Table 1:** Summary of patient characteristics for Vestibular Migraine group and Vestibular Dysfunction group.

	Group	
Characteristics of subjects	VM	VD
Number	30	30
Mean age (years)	46.13	56.50
Age range	30–64	38–76
Sex (male/female)	2/28	10/20
Time since onset of vestibular problems (months–mean)	9.3	10.2
Subjects with abnormal VHIT results (%)	33	35
Subjects with abnormal VNG results (%)	81	75

VM, Vestibular Migraine group; VD, Vestibular Dysfunction group; VHIT, Video Head Impulse Test; VNG, Videonystagmography.

Abnormal findings were found in at least one vestibular test in 81% of the patients with VM and 75% of the patients with VD. Our results are similar to the literature.^17^, ^20^, ^21^

**Figure 1:**
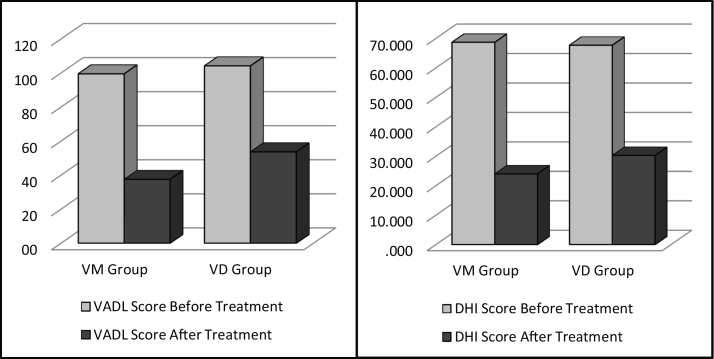
Pre- and post-treatment DHI and VADL scores in VM and VD groups. (DHI: dizziness handicap inventory, VADL: vestibular disorders activities of daily scale, VM, Vestibular Migraine group; VD, Vestibular Dysfunction group).

**Figure 2:**
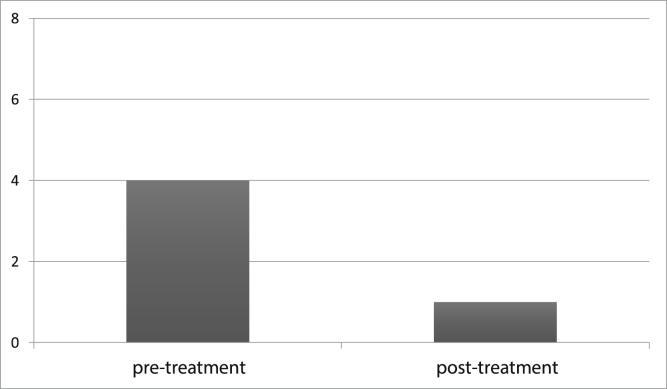
Comparison of headache scores between pre- and post-treatment in Vestibular Migraine Group.

To document the effectiveness of VR in both groups, subjective self-report measurements (DHI and VADL scores) were taken at the beginning and end of the vestibular rehabilitation treatment. Changes in DHI and VADL scores are given in Table 2. Before treatment, the mean DHI score was 68.7, and VADL score was 99.5 in the VM group, while it was 24.0 and 37.5, respectively, after treatment. In the VD Group, it was found to be 67.7 (DHI score) and 104.2 (VADL score) before treatment and 30.3 (DHI score) and 53.7 (VADL score) after treatment (Table 2 and Fig. 1). In VM Group; mean pre-treatment frequency of dizziness 3, and mean post-treatment frequency was 1, mean pre-treatment frequency of headache was 4, and mean post-treatment frequency of headache was 1. In VD Group; mean pre-treatment frequency of dizziness was 3, and mean post-treatment frequency of dizziness was 1. Accordingly, dizziness frequency decreased from 3 to 1 with treatment in the VM Group, while the frequency of headache decreased from 4 to 1 on average (Fig. 2). For the VD group, the frequency of dizziness decreased from 3 to 1. Results show that the DHI score, VADL score, and the frequency of dizziness and headache scores significantly improved with VR in both the VM Group and the VD Group (*p* < 0.05).

**Table 2:** Pre- and post-treatment mean DHI and VADL scores in VM and VD groups.

	VM Group		VD Group		*p*
	Mean ± SD	median	Mean ± SD	median	
DHI Score					
Before treatment	68.7 ± 8.5	66.0	67.7 ± 19.5	62.0	0.654^a^
After treatment	24.0 ± 8.8	20.0	30.3 ± 23.0	20.0	0.881^a^
Intra group difference	0.000^b^		0.000^b^		
VADL Score					
Before treatment	99.5 ± 10.7	99.5	104.2 ± 50.6	87.0	0.024^a^
After treatment	37.5 ± 8.6	37.5	53.7 ± 44.0	40.5	1.000^a^
Intra group difference	0.000^b^		0.000^b^		

DHI, Dizziness Handicap Inventory; VADL, Vestibular Disorders Activities of Daily scale, SD, Standard Deviation; VM, Vestibular Migraine group; VD, Vestibular Dysfunction group.

a Mann-Whitney U test.

b Wilcoxon test.

Pre- and post-treatment CD condition values are shown in Table 3, Table 4, Table 5, Table 6. For the VM Group, the pre- and post-treatment SOT results are shown in Table 3, Table 4. SOT results for VD Group are given in Table 5, Table 6. For both groups, condition values were higher after treatment, except mediolateral C2 score in VD Group. Pre- and post-treatment SOT 1–5 values (somatosensorial, visual, vestibular, preferential, and global scores) are shown in Table 7, Table 8. Post-treatment results are higher than pre-treatment results for both groups (except the mediolateral vestibular and preferential scores in the VM Group and anteroposterior global score in the VD Group).

**Table 3:** Posturography values for SOT conditions (anteroposterior) – VM Group.

Condition	Pre-treatment			post-treatment			*p*
	Mean ± SD	Median	Range (min–max)	Mean ± SD	Median	Range (min-max)	
C1	74.2 ± 13.9	74.0	52.7–93.9	91.9 ± 0.8	92.3	90.8–92.7	0.000^a^
C2	62.8 ± 18.9	61.0	24.3–91.3	88.6 ± 0.7	88.6	87.7–89.3	0.000^a^
C3	48.2 ± 29.0	48.0	00.0–90.5	80.3 ± 4.8	81.9	73.9–85.0	0.000^a^
C4	49.6 ± 23.6	55.9	00.0–77.0	76.5 ± 0.8	77.0	75.4–77.1	0.000^a^
C5	32.8 ± 15.4	32.7	00.0–61.2	65.0 ± 5.5	63.6	59.5–72.4	0.000^a^
C6	8.4 ± 13.4	0.0	00.0–48.7	32.3 ± 15.7	32.9	13.1–50.8	0.000^a^

SOT, Sensory Organization Test; C, Condition; SD, Standard Deviation; VM, Vestibular Migraine group.

a Wilcoxon test.

**Table 4:** Posturography values for SOT conditions (mediolateral) – VM Group.

Condition	Pre-treatment			Post-treatment			*p*
	Mean ± SD	Median	Range (min–max)	Mean ± SD	Median	Range (min–max)	
C1	84.2 ± 16.3	90.0	31.6–96.7	93.2 ± 3.5	92.3	89.4–97.8	0.002^a^
C2	74.1 ± 25.5	85.4	9.8–94.8	88.3 ± 7.1	91.9	85.0–97.9	0.021^a^
C3	72.1 ± 17.5	71.5	37.9–95.6	86.3 ± 6.2	88.4	78.5–94.5	0.000^a^
C4	51.7 ± 14.9	52.6	28.3–79.0	66.1 ± 7.1	64.0	58.5–75.6	0.000^a^
C5	27.9 ± 15.8	28.0	00.0–50.4	55.2 ± 12.6	63.2	37.8–64.7	0.000^a^
C6	11.7 ± 14.8	6.8	00.0–50.5	30.8 ± 0.7	30.4	30.2–31–7	0.000^a^

SOT, Sensory Organization Test; C, Condition; SD, Standard Deviation; VM, Vestibular Migraine group.

a Wilcoxon test.

**Table 5:** Posturography values for SOT conditions (anteroposterior) – VD Group.

Condition	Pre-treatment			Post-treatment			*p*
	Mean ± SD	Median	Range (min–max)	Mean ± SD	Median	Range (min–max)	
C1	73.5 ± 10.8	73.5	48.7–91.4	82.9 ± 9.3	85.3	70.5–93.2	0.001^a^
C2	64.3 ± 19.1	64.2	17.7–89.5	78.4 ± 15.9	81.5	39.3–94.9	0.003^a^
C3	49.9 ± 21.3	48.0	00.0–80.7	65.1 ± 21.5	70.9	16.1–85.6	0.002^a^
C4	57.5 ± 10.7	57.5	34.1–78.1	69.5 ± 9.5	70.0	50.3–83.1	0.000^a^
C5	40.3 ± 14.9	40.1	2.4–65.0	52.7 ± 15.4	52.8	26.1–74.8	0.004^a^
C6	16.3 ± 14.4	16.3	00.0–45.7	34.6 ± 17.4	38.0	00.0–59.1	0.001^a^

SOT, Sensory Organization Test; C, Condition; SD, Standard Deviation; VD, Vestibular Dysfunction group.

a Wilcoxon test.

**Table 6:** Posturography values for SOT conditions (mediolateral) – VD Group.

Condition	Pre-treatment			Post-treatment			p
	Mean ± SD	Median	Range (min–max)	Mean ± SD	Median	Range (min–max)	
C1	83.8 ± 12.1	83.8	48.00–97.80	92.1 ± 4.2	92.6	85.3–97.7	0.000^a^
C2	80.3 ± 14.7	80.2	21.50–96.50	85.0 ± 11.6	87.8	58.3–95.9	0.106^a^
C3	73.5 ± 17.8	73.5	00.0–95.60	84.3 ± 9.1	84.8	62.6–97.0	0.001^a^
C4	52.4 ± 16.9	52.8	00.0–75.90	64.1 ± 15.6	65.5	30.1–79.8	0.004^a^
C5	28.4 ± 12.8	27.0	00.0–51.80	43.7 ± 15.1	42.7	25.9–71.3	0.001^a^
C6	10.2 ± 13.4	10.1	00.0–57.20	33.7 ± 17.0	34.1	00.0–65.1	0.000^a^

SOT, Sensory Organization Test; C, Condition; SD, Standard Deviation; VD, Vestibular Dysfunction group.

a Wilcoxon test.

**Table 7:** The results of the Sensory Organization Test (SOT) in the pre- and post-treatment periods (VM group).

	Pre-treatment (AP)			Post-treatment (AP)			*p*
	Mean ± SD	Median	Range	Mean ± SD	Median	Range	
Somatosensorial score	78.7 ± 14.7	82.0	46.2–90.0	90.0 ± 0.0	90.0	88.1–92.5	0.000^a^
Visual score	66.2 ± 20.8	77.0	25.3–84.1	82.7 ± 1.9	84.0	80.0–84.2	0.000^a^
Vestibular score	44.8 ± 16.0	46.0	11.2–62.0	59.7 ± 3.4	62.0	55.2–62.1	0.000^a^
Preferential score	58.1 ± 20.8	65.0	19.4–77.0	68.7 ± 10.6	75.0	54.3–77.0	0.042^a^
Global score	47.1 ± 11.5	48.0	25.5–63.1	63.0 ± 2.9	65.0	59.4–65.0	0.000^a^

	Pre-treatment (ML)			Post-treatment (ML)			*p*
	Mean ± SD	Median	Range	Mean ± SD	Median	Range	
Somatosensorial score	85.7 ± 17.4	96.0	37.1–97.1	97.0 ± 0.0	98.0	97.0–97.0	0.000^a^
Visual score	59.2 ± 15.5	62.0	26.2–75.0	67.7 ± 7.8	71.0	57.1–75.0	0.038^a^
Vestibular score	39.9 ± 17.4	41.0	00.1–55.4	46.3 ± 12.5	53.0	29.2–55.1	0.263^a^
Preferential score	68.3 ± 7.7	70.0	48.0–74.1	70.7 ± 4.8	74.0	64.5–74.1	0.388^a^
Global score	52.5 ± 8.9	52.0	39.1–67.0	62.0 ± 5.2	64.0	55.0–67.2	0.000^a^

AP, Anteroposterior; ML, Mediolateral; SD, Standard Deviation; VM, Vestibular Migraine group.

a Wilcoxon test.

**Table 8:** The results of the Sensory Organization Test (SOT) in the pre and post-treatment periods (VD Group).

	Pre-treatment (AP)			Post-treatment (AP)			*p*
	Mean ± SD	Median	Range	Mean ± SD	Median	Range	
Somatosensorial score	82.3 ± 9.4	82.0	61.1–90.0	87.1 ± 7.9	90.0	64.1–90.2	0.025^a^
Visual score	75.1 ± 8.9	75.0	50.2–84.1	81.1 ± 6.0	84.0	64.0–84.4	0.007^a^
Vestibular score	49.9 ± 14.7	51.0	7.2–62.5	57.9 ± 10.3	62.0	58.0–63.2	0.008^a^
Preferential score	52.9 ± 5.8	55.0	26.4–77.4	61.6 ± 15.7	65.0	26.1–77.1	0.007^a^
Global score	52.0 ± 10.1	53.0	25.2–69.0	53.7 ± 11.0	54.0	34.2–69.0	0.531^a^

	Pre-treatment (ML)			Post-treatment (ML)			*p*
	Mean ± SD	Median	Range	mean ± SD	Median	Range	
Somatosensorial score	93.3 ± 4.2	94.0	82.1–97.0	95.8 ± 2.8	97.0	88.0–97.1	0.003^a^
Visual score	61.6 ± 13.9	60.0	31.0–75.1	72.3 ± 3.2	73.5	68.1–75.2	0.000^a^
Vestibular score	36.5 ± 14.1	37.0	00.1–55.4	45.8 ± 8.4	47.5	33.1–55.0	0.006^a^
Preferential score	68.5 ± 10.5	70.0	21.1–74.4	72.5 ± 2.4	73.5	66.2–74.0	0.011^a^
Global score	53.5 ± 8.6	53.5	24.0–67.3	58.1 ± 8.8	58.5	40.1–67.1	0.040^a^

AP, Anteroposterior; ML, Mediolateral; SD, Standard Deviation; VD, Vestibular Dysfunction group.

a Wilcoxon test.

## Discussion

Today, VR is an accepted treatment method for patients with balance and vestibular problems. Vestibular rehabilitation, which has been used in the treatment of balance disorders since the 1940s, is an exercise-based treatment method, and aimed to optimize vestibular compensation.^22^, ^23^, ^24^ Cochrane studies and clinical practice guidelines have reported that VR is a safe and effective treatment method for these patients.^22^, ^25^ VR is particularly useful in patients with nonspecific dizziness and increased risk of falls. VR is a physiological therapy method that acts on the vestibular system to stimulate CNS plasticity, supports the restoration of body balance, accelerates the mechanisms of compensation, adaptation, and habituation. It has been suggested that VR increases the quality of life in individuals with dizziness and balance disorder.^26^ The leading VR principles are desensitizing the vestibular system, increasing vestibulo-ocular and vestibulo-spinal reflex gains, and creating new alternative senses against imbalance triggered by position change. Improvement in all these mechanisms results in progressive improvement in dizziness and vertigo.^27^

Components of vestibular rehabilitation include “desensitizing” the vestibular system by provoking symptoms, learning to coordinate eye and head movements, developing balance, and walking skills, and learning to handle disturbing situations. VR components: 1) Compensation/habituation; 2) Adaptation (an adaptation of VOR, gaze stabilization), 3) Sensory substitution (substitution of other strategies for lost function), 4) Motor learning to change movement behavior includes postural control exercises, fall prevention, relaxation exercises, reconditioning exercises, and functional retraining.^28^, ^29^, ^30^, ^31^

VR restores homeostasis in the vestibular system, and these adaptation mechanisms allow the treatment of symptoms and a stable posture in the long term. A Cochrane Database Systematic Review published in 2015 concluded that there is moderate to strong evidence supporting VR in managing patients with vestibular hypofunction for reducing symptoms and improving function.^25^ Therapeutic exercises targeting functional limitations and symptoms caused by vestibular insufficiency reduce dizziness, decrease the risk of falling by increasing postural stability, and increase visual acuity during head movements.

A systematic review concluded that there is moderate evidence to support the effectiveness of vestibular exercise in individuals with bilateral vestibular hypofunction for improving gaze and postural stability.^32^ Vestibular exercises have been shown to be effective compared to no or placebo exercises.^22^ Studies support the view that VR is effective in peripheral vestibulopathy compared with placebo, sham, or no intervention.^33^ Giray et al. divided 42 patients with chronic vestibular dysfunction into two groups (VR Group and control group) and applied VR to only VR Group, and reported that all assessment scales (symptoms, disability, balance, and postural stability) were better in the VR Group.^34^ VR improves balance skills and increases self-confidence, increasing patient's activity and quality of life.^35^, ^36^, ^37^

Wrisley et al. retrospectively evaluated 30 patients with vestibular impairment with or without migraine headache who treated with VR. They applied general strengthening and stretching exercises, canalit repositioning technique, exercises using different sensory inputs to increase balance control to these patients.

They reported that both groups of patients benefited clinically from VR. Recovery occurred in 88% of patients with migraine and 89% of patients with non-migraine.^16^ Vitkovic et al. evaluated the effect of VR in dizzy patients with and without migraine. In both subjective and objective measurements, VM patients benefited the same as the other patient group.^15^ Whitney et al. investigated the effect of physical therapy on VM patients enrolled in VR program, using a retrospective chart review. A rehabilitation treatment designed for each patient was applied. In patients with migraine headache, a significant improvement was reported in all measurements with VR treatment.^17^

Sugaya et al. worked with 251 dizzy patients (28 V M, 144 without any headache, 79 patients with tension-type headache). The exercise program included repeated training of the vestibulo-ocular reflex and vestibulo-spinal reflex. They reported significant improvement with VR in headache, dizziness, and physiological scores (DHI, HIT, hospital anxiety, and depression scale) in patients with VM and tension-type headache group. Although there was a significant improvement in dizziness and physiological scores in patients with headache, headache scores increased after a period of treatment. Sugaya et al. explain this with the increased awareness of other symptoms (e.g., headache) as dizziness improves.^14^ Alghadir and Lauritsen reported retrospective studies stating that VR is effective in VM.^28^, ^38^ Shaabani et al. found a significant improvement in dizziness and headache and a significant reduction in DHI and VADL problems in their studies. Shaabani et al. think that these results show the effect of VR on balance problems.^39^ Gottshall et al. performed specific exercises to reduce dizziness, increase balance, and improve overall activity levels in patients with migraine-associated dizziness. They reported a statistically significant improvement in DHI scores, balance confidence score, dynamic gait index, and dynamic posture score.^40^ Hansson et al. reported that VR is effective in reducing vestibular complaints and headaches in VM.^41^

Various outcome measurement methods are used to evaluate the effect of VR on treatment (frequency of dizziness – vertigo, postural instability, gait, risk of fall, quality of life).^42^, ^43^ Self-report is a powerful measurement method for people with dizziness. In general, if a person feels better and is more involved in life events, that person is considered to be recovering. DHI and VADL were used in our study. DHI is the most used method, and it measures the perception of disability against dizziness.

Another measurement method is VADL, which measures the effect of dizziness or vestibular dysfunction on primary activities in daily life. In our patients in both the VM and VD groups, it was observed that both evaluation scores improved with VR treatment. Similarly, the frequency of dizziness, a subjective measurement method, decreased in both groups of patients. SOT values were used as objective measurement methods, and significant improvement was observed in SOT values in our VR patients after treatment.

In migraine, patients are more sensitive to vestibular stimuli because their sensitivity to external stimuli increases, so their perception of vestibular dysfunction symptoms may be increased.^44^, ^45^ Vitkovic et al. think that patients with VM perceive their symptoms more seriously than patients with VD, although they have a similar peripheral vestibular function, physical performance, and symptom duration.^15^ In migraine, perception and behavioral thresholds change due to altered central excitability. The cortical hyperexcitability theory has also been supported by electrophysiological and magnetic stimulation studies.^46^ As a result, patients with migraine have a stronger reaction to intense, repetitive, or prolonged stimulation. This altered sensory modulation may be due to increased neural excitability or decreased neural inhibition at the synaptic or intrinsic neuronal level. VR helps create new balance patterns and reduce dizziness complaints by correcting vestibular functions and compensating for defects. Therefore, the auxiliary role of VR in the optimal treatment of VM is accepted.^4^, ^14^, ^15^, ^38^

In our study, patients with vestibular problems with or without migraine were compared, and no treatment or placebo group was included. Because it is considered unethical to have “no treatment” or a placebo control group. Nevertheless, studies with a control group should be conducted to evaluate the positive effect of VR in the long run.

## Conclusion

In patients with vestibular problems with or without migraine who underwent VR, a significant improvement was observed in subjective and objective balance assessment measurements after treatment. VR should definitely be considered in patients who do not or limited benefit from medical therapy.

## Conflicts of interest

The authors declare no conflicts of interest.
